# Retrospective Metabolomics Profiling of Clinical Urine Drug Screen Samples Reveals Features Associated with Opiate Exposure

**DOI:** 10.3390/metabo16080558

**Published:** 2026-08-06

**Authors:** Delaney Morrow, Rachel K. Vanderschelden, Kenichi Tamama

**Affiliations:** 1 Department of Pathology, University of Pittsburgh School of Medicine; Pittsburgh, PA 15261, USA; morrow.delaney@medstudent.pitt.edu (D.M.); rachelkvds@gmail.com (R.K.V.); 2 Clinical Laboratories, University of Pittsburgh Medical Center Presbyterian Hospital, Pittsburgh, PA 15213, USA; 3 McGowan Institute for Regenerative Medicine, University of Pittsburgh, Pittsburgh, PA 15219, USA

**Keywords:** opiate, urine drug screening, metabolomics, biomarkers, liquid chromatography–mass spectrometry, opioid-related disorders, heroin, oxycodone, retrospective studies

## Abstract

**Background/Objectives:** Opiates comprise naturally occurring opium alkaloids and their semisynthetic derivatives. Routine urine drug screening relies on enzyme immunoassays (EIAs) to rapidly detect opiate exposure; however, EIAs provide limited insight into opiate-associated metabolic patterns. **Methods:** We retrospectively analyzed liquid chromatography–quadrupole time-of-flight mass spectrometry (LC-qToF-MS) datasets from comprehensive urine drug screening of 363 patients at the University of Pittsburgh Medical Center Clinical Toxicology Laboratory. Multiple statistical analyses were applied to identify the features associated with opiate (OPIA)-EIA-positive, oxycodone (OXY)-EIA-positive, and 6-monoacetylmorphine (6MAM)-EIA-positive specimens (42, 34, and seven specimens, respectively) designated as EIA-associated discovery feature sets. The feature sets selected by ≥2 statistical analyses were defined as EIA-associated consensus feature set and further evaluated using MS-FINDER for feature annotation. **Results:** Among 14,883 features, 138, 121, and 104 features were assigned to the OPIA-, OXY-, and 6MAM-EIA discovery feature sets, respectively. Consensus feature sets included oxycodone/opiate metabolites, acetaminophen metabolites, and norfentanyl for OPIA-EIA; oxycodone metabolites, α-phenylalanylaspartic acid, and 4-pyridoxic acid for OXY-EIA; and norfentanyl, 6-monoacetylmorphine, and 3-hydroxycotinine artifact for 6MAM-EIA. **Conclusions:** These metabolomic patterns indicate a dominant exposure gradient model, in which OXY-EIA-positive specimens primarily reflect prescribed oxycodone exposure, 6-MAM-EIA-positive specimens reflect illicit heroin/fentanyl exposure with polysubstance/recreational use signature, and OPIA-EIA-positive specimens occupy an intermediate, mixed profile shaped by immunoassay cross-reactivity and real-world co-exposures. Associations involving α-phenylalanylaspartic acid and 4-pyridoxic acid are hypothesis-generating and require further validation. These findings illustrate the value of archived clinical toxicology datasets for metabolomic discovery and as a foundation for sentinel laboratory-based surveillance of evolving drug and chemical exposures.

## 1. Introduction

Opiates are prototypal opioids with a long history of use and abuse [1,2,3]. They are naturally occurring opium alkaloids and their semisynthetic derivatives. Morphine is the principal opiate alkaloid in *Papaver somniferum*, whereas heroin, or 3,6-diacetylmorphine, is a diacetylated morphine derivative and oxycodone is a semisynthetic derivative of thebaine, another opium alkaloid [4,5,6]. Their shared morphinan/phenanthrene scaffold contributes to opioid receptor binding [6] and, in part, to the antibody cross-reactivity exploited by the immunoassays targeting opiates, although each assay specificity varies [7].

In the United States, positive opiate-related immunoassay results may reflect prescribed medications, prescription-diverted oxycodone, illicit opiates such as heroin, or related metabolites [8,9]. Conventional immunoassays are useful for rapid screening but only provide limited information about co-exposures, downstream metabolic changes, and endogenous biochemical responses.

In contrast, untargeted metabolomics can address this limitation by surveying a broad range of xenobiotic and endogenous features in clinical specimens. The growing adoption of liquid chromatography–high-resolution mass spectrometry (LC-HRMS), including liquid chromatography–quadrupole time-of-flight mass spectrometry (LC-qToF-MS), has expanded the role of untargeted acquisition in clinical toxicology [10,11,12,13].

As part of routine clinical care, the Clinical Toxicology Laboratory at the University of Pittsburgh Medical Center performs comprehensive urine drug screening (CUDS; >500 specimens per month) to evaluate suspected intoxication and medication adherence. This LC-qToF-MS workflow, with an all-ion fragmentation scan, generates untargeted mass spectral data across a broad range of detected analytes [9,11,14,15]. Although each specimen typically contains thousands of molecular features, routine clinical interpretation focuses on a limited set of known xenobiotics, primarily drugs and their metabolites. As a result, most detected features remain unannotated and are not incorporated into routine reporting. These residual feature-level data represent an underused resource for investigating co-exposures, drug-associated metabolic perturbations, and broader biochemical signatures linked to drug exposure.

In this study, we repurposed data generated through our CUDS workflow, which uses LC-qToF-MS for qualitative toxicological testing [11]. Although this platform is primarily intended for routine drug detection, its untargeted acquisition produces feature-rich datasets suitable for secondary metabolomics analysis. Using archived mass spectrometry data, we performed a retrospective metabolomics study in which specimens were classified based on qualitative opiate, oxycodone, and 6-monoacetylmorphine enzyme immunoassay (OPIA-EIA, OXY-EIA, and 6MAM-EIA, respectively) results as metadata. By comparing EIA-positive and EIA-negative specimens, we sought to characterize the distinct and overlapping metabolomic signatures associated with opiate-related immunoassay positivity.

This study also has translational significance because it repurposes data generated from a clinically accessible specimen type and an analytical workflow already embedded in routine toxicology practice. Compared with metabolomics studies using specimens that are difficult to obtain or implement clinically, such as brain tissue homogenates, urine-based CUDS data provide a more practical bridge between discovery and clinical validation. Features identified in this setting may therefore be prioritized as candidate biomarkers, co-exposure indicators, or interpretive adjuncts for future testing, while also supporting laboratory-based sentinel surveillance for evolving drug exposures and emerging adulterants.

## 2. Materials and Methods

### 2.1. Specimens

The CUDS datasets were originally derived from the urine specimens from 363 patients, as described previously [11] (Table 1). These urine specimens were from emergency departments of UPMC hospitals and clinics in western PA. These specimens underwent the initial EMIT-II-based qualitative drug screening panel including for opiate (OPIA-EIA, with the cutoff at 300 ng/mL for morphine), oxycodone (OXY-EIA, with the cutoff at 100 ng/mL for oxycodone), and 6-monoacetylmorphine (6MAM-EIA, with the cutoff at 20 ng/mL for 6-monoacetylmorphine) (Siemens Healthineers USA, Malvern, PA, USA) before mass spectrometry-based analysis. OPIA-EIA can detect opiates and their metabolites broadly (e.g., 6-monoacetylmorphine at 435 ng/mL, codeine 102–306 ng/mL, heroin 396 ng/mL, hydrocodone at 247 ng/mL, hydromorphone at 498 ng/mL, morphine-3-glucuronide at 626 ng/mL, nor-oxycodone at 100 µg/mL, oxycodone at 1500 ng/mL, and oxymorphone 9300 ng/mL, according to the product insert). One volume of urine specimen was mixed with 4 volumes of distilled water spiked with nitrazepam and tenoxicam as internal standards (40 ng/mL final), and 3 µL of the diluted specimen was injected into the LC-qToF-MS system.

### 2.2. LC-qToF-MS Assay Conditions/Settings in CUDS

A dilute-and-shoot method with 1:4 volume mixture was employed for LC-qToF-MS analysis in positive electrospray ionization (ESI) mode. The acquired high-resolution mass spectrometry (HRMS) datasets were initially analyzed using UNIFI^®^ Scientific Information System (Waters, Milford, MA, USA) to screen compounds through retention time, monoisotopic mass accuracy, and fragment ions match and the CUDS reports with identified drugs were released as CUDS reports as part of clinical testing previously. Detailed information for LC-qToF-MS assay conditions was given previously [11,14].

### 2.3. Overall Data Processing and Analytical Workflow

**Figure 1:**
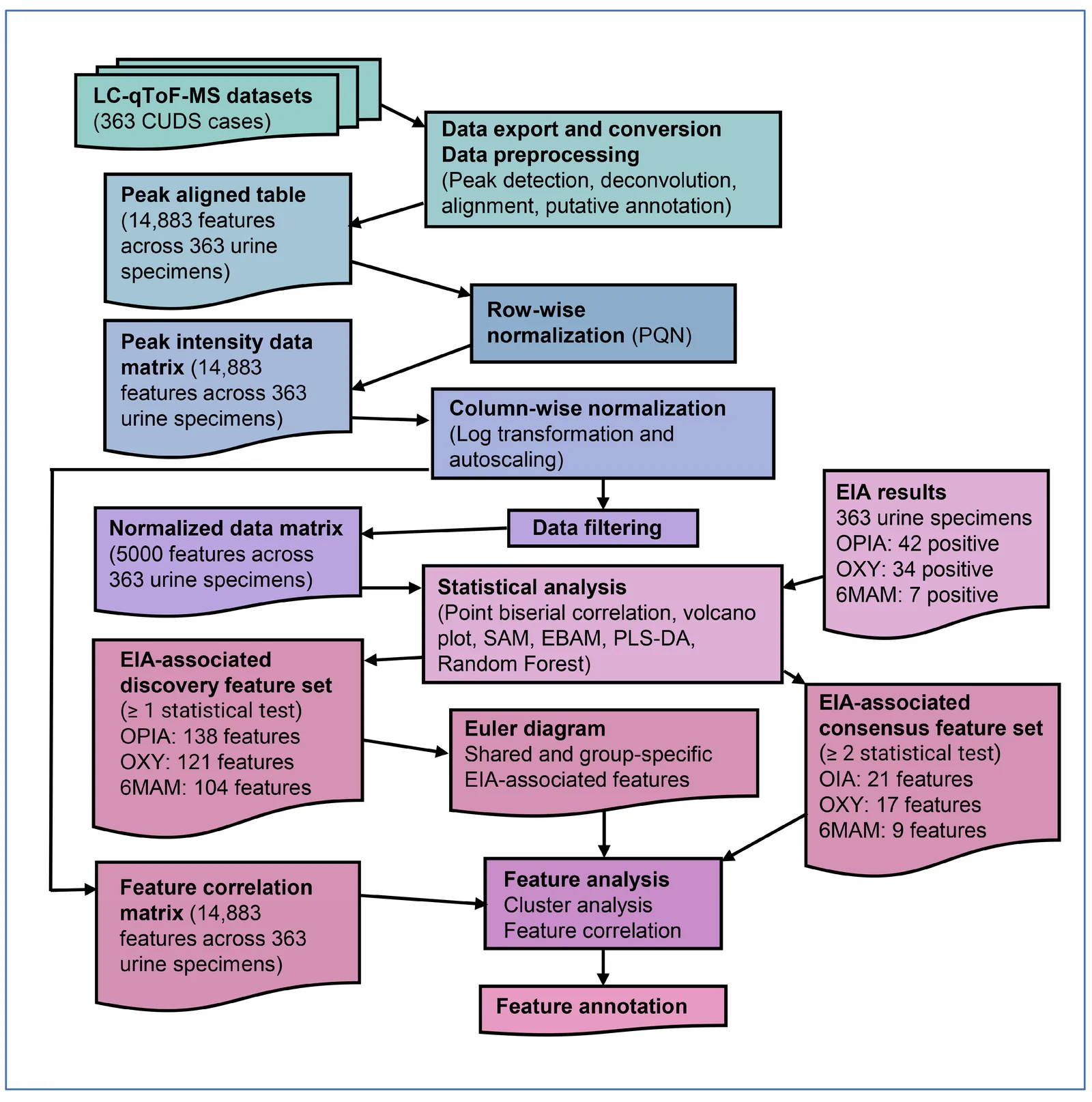
Analytical and computational workflow for untargeted urine metabolomics analysis of opiate-associated features.

The overall data processing and analysis workflow, from raw mass spectrometry data to metabolomics feature annotation, is summarized in Figure 1. Detailed information for overall data processing and analytical workflow was described previously [11].

#### 2.3.1. Data Processing and Statistical Analysis

De-identified MS datasets in (.uep) files were exported, converted to (mzML) files, and processed using MS-DIAL version 4.9 [16] for peak detection and peak alignment. The peak intensity matrix was normalized row-wise by probabilistic quotient normalization (PQN), followed by log transformation, autoscaling and unsupervised data filtering to reduce the dataset to 5000 features for the following statistical analyses by MetaboAnalyst (web version and MetaboAnalystR) [17,18,19,20,21].

Point-biserial correlation, volcano plot analysis, significance analysis of microarrays and metabolites (SAM), empirical Bayesian analysis of microarrays and metabolites (EBAM), partial least-squares discriminant analysis (PLS-DA), and random forest (RF) were applied independently to the OPIA-, OXY-, and 6MAM-EIA outcomes to identify metabolic features significantly associated with these EIA results. Point-biserial correlation features were selected at a false discovery rate (FDR) of <0.05. Volcano plot analysis (fold change ≥ 2, *p* < 0.05), SAM and EBAM evaluated FDR-controlled significance (EBAM posterior delta, 0.9; FDR < 0.05), and PLS-DA feature selection was retained only when 2000-permutation testing was statistically significant. RF was performed after class balancing by combined oversampling of the minority class and undersampling of the majority class using the ROSE package (0.0-4), as described previously [11].

**Figure 2:**
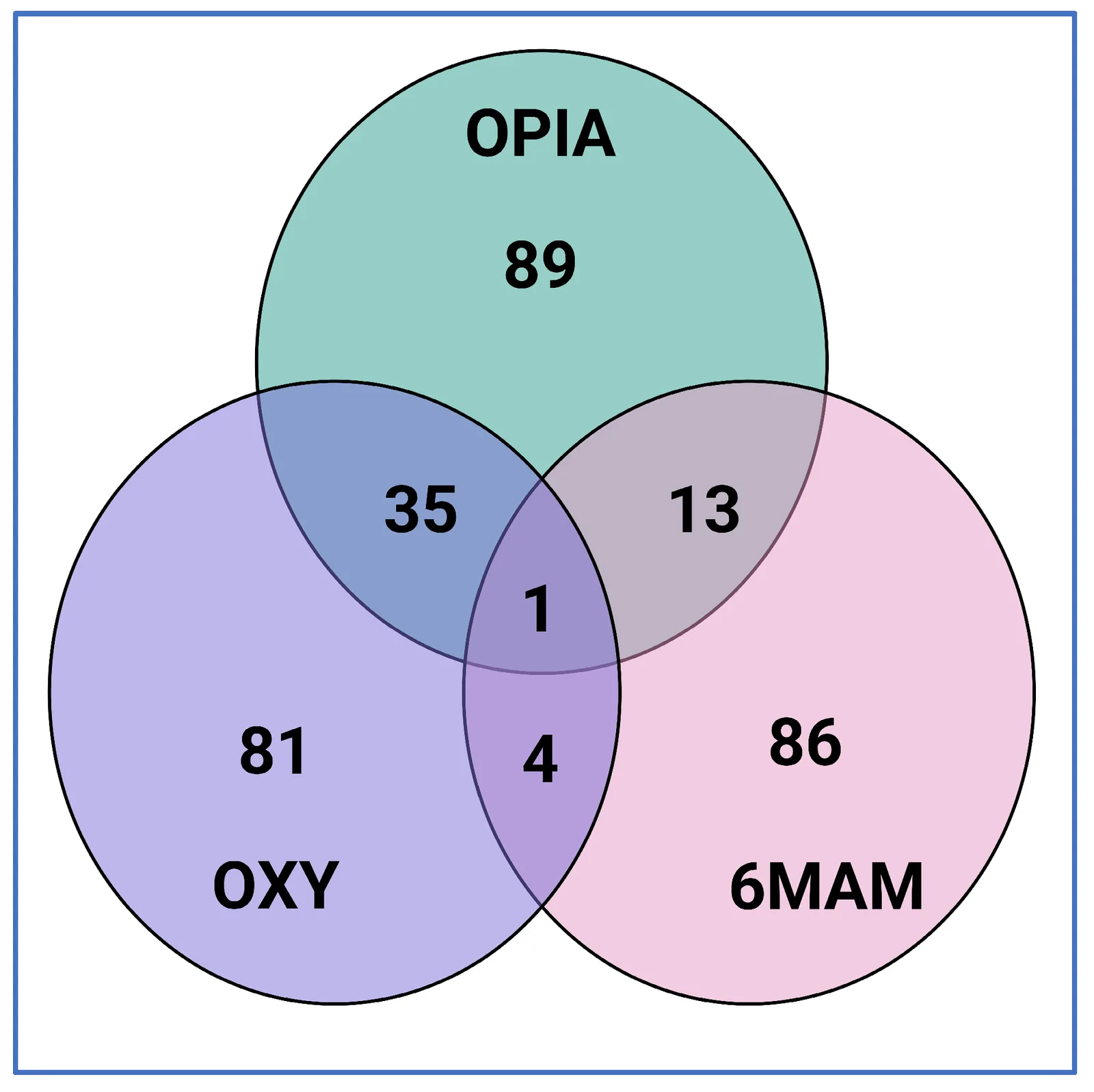
Overlap among OPIA-, OXY-, and 6MAM-associated discovery feature sets. Euler diagram showing unique and shared features for each discovery feature set. Most features were specific to one discovery set, with smaller subsets shared between two or all three groups, consistent with distinct but partially overlapping metabolic signatures across the opiate–oxycodone–heroin exposure continuum.

The features selected by ≥1 statistical analysis were designated as discovery feature sets, whereas the features selected by ≥2 statistical analyses were designated as consensus feature sets. Because the statistical methods used different selection and error control criteria, the consensus requirement was used as a cross-method robustness filter and not as a substitute for formal multiple-testing correction. A Euler diagram was generated from the discovery feature sets to summarize shared and group-specific features across EIA-defined groups (Figure 2, Table S1).

#### 2.3.2. Correlation-Based Hierarchical Clustering Within EIA-Associated Discovery Feature Sets

**Figure 3:**
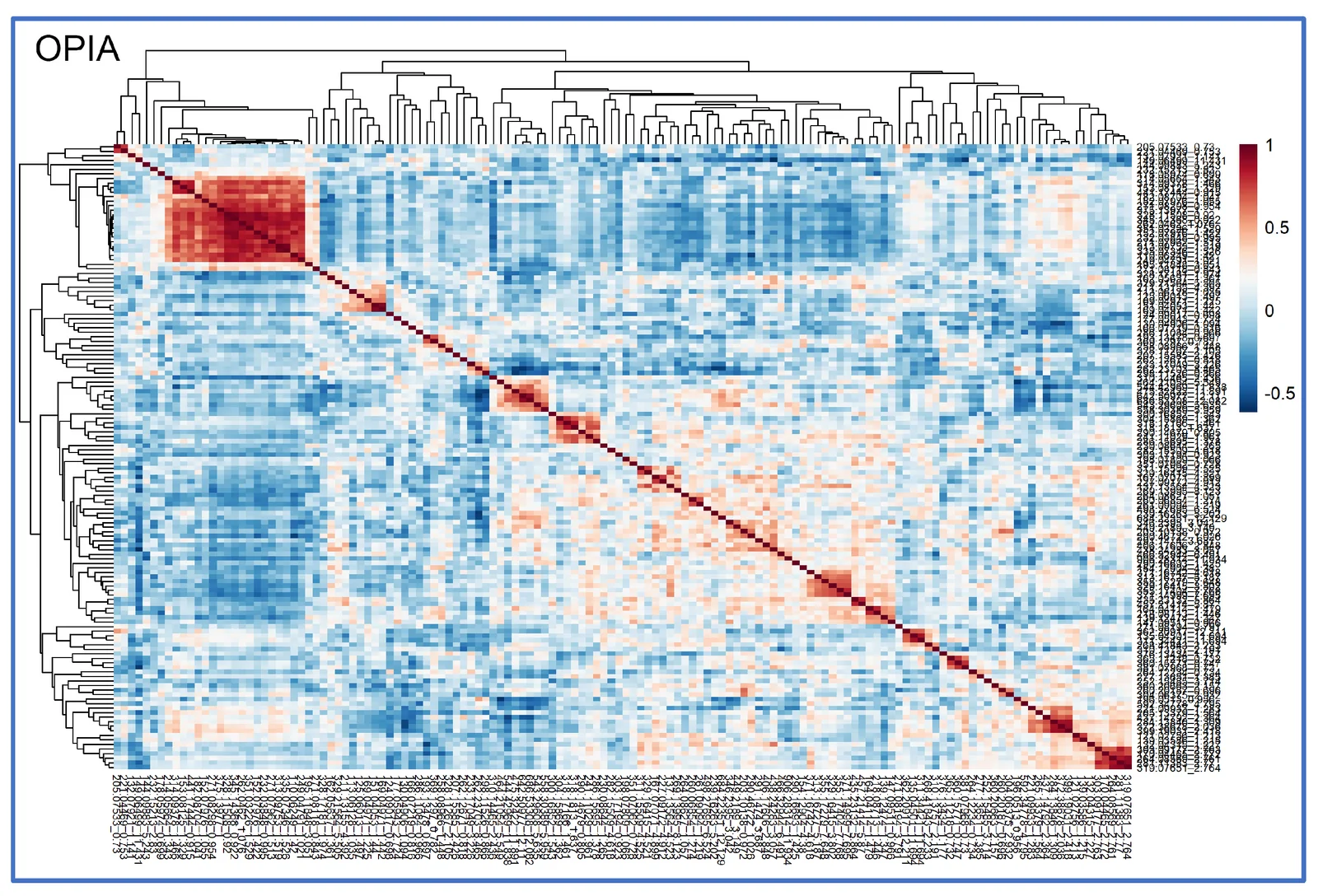
Correlation heatmap and dendrogram of the associated features for 138 OPIA-EIA-associated features. High-level dendrogram slicing (k = 7) defined Clusters A–G, middle-level slicing (k = 14) defined subclusters, and low-level slicing (k = 50) defined terminal branches used in Table S2.

**Figure 4:**
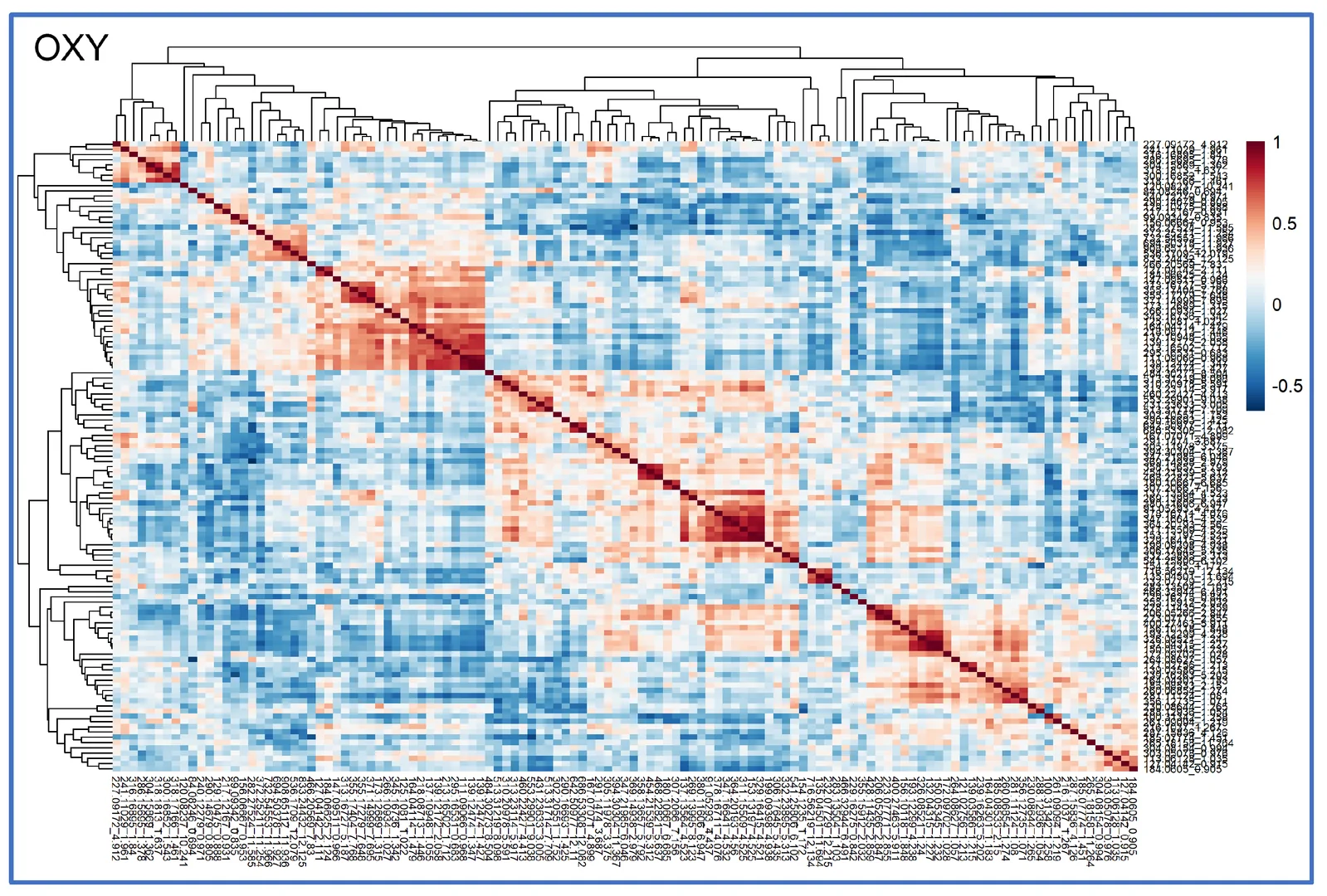
Correlation heatmap and dendrogram of the associated features for 121 OXY-EIA-associated features. High-level dendrogram slicing (k = 7) defined Clusters A–G, middle-level slicing (k = 14) defined subclusters, and low-level slicing (k = 50) defined terminal branches used in Table S2.

**Figure 5:**
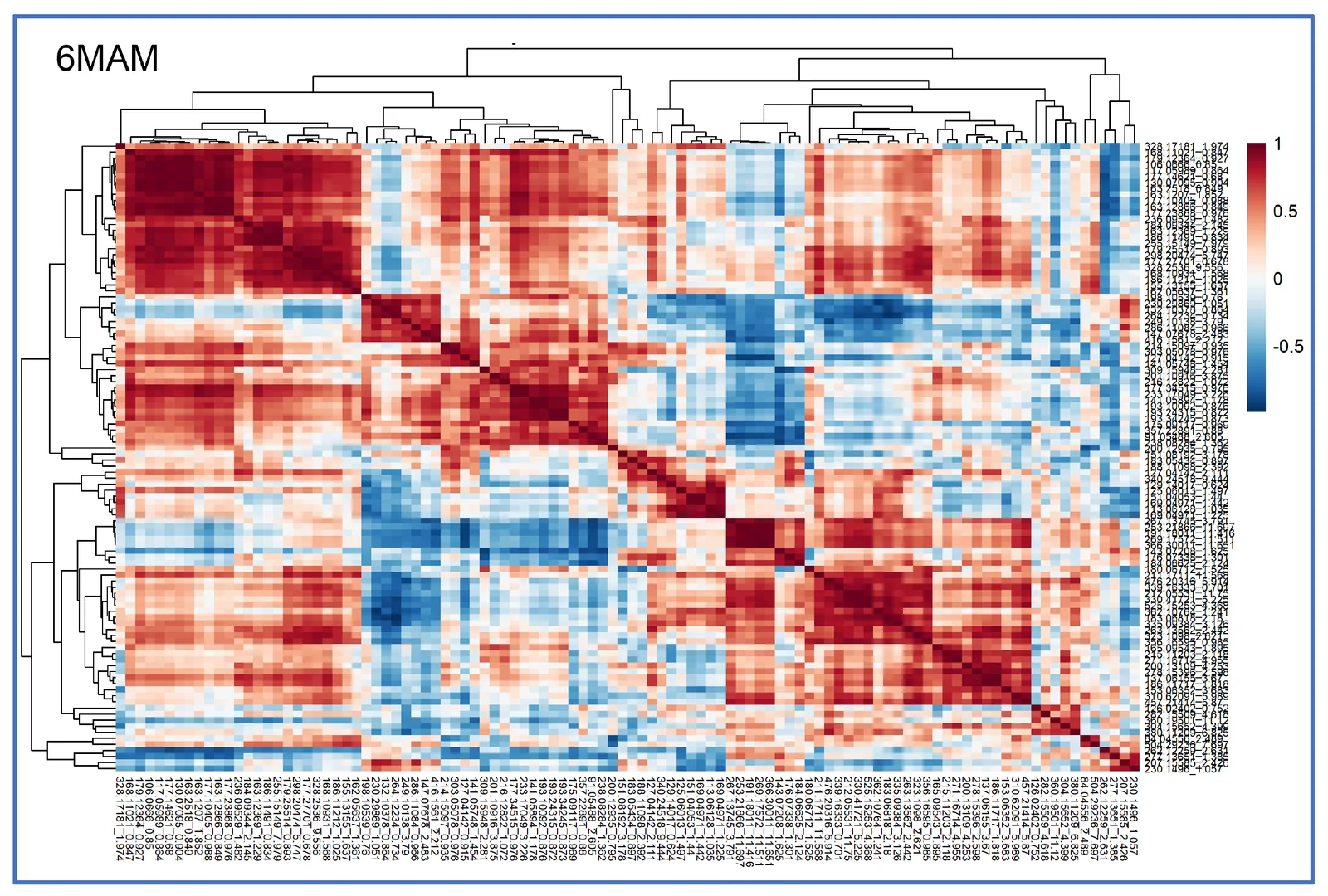
Correlation heatmap and dendrogram of the associated features for 104 6MAM-EIA-associated features. High-level dendrogram slicing (k = 7) defined Clusters A–G, middle-level slicing (k = 14) defined subclusters, and low-level slicing (k = 50) defined terminal branches used in Table S2.

Hierarchical correlation clustering analysis was conducted by computing Pearson correlation coefficients between the features within the EIA-associated discovery feature set. The resulting matrix was visualized as a heatmap using the pheatmap package (1.0.13) in R and hierarchical clustering was performed using Ward’s method. Features were grouped into seven clusters (Clusters A–G) by high-level dendrogram slicing (k = 7). Additionally, middle-level (k = 14) and low-level (k = 50) dendrogram slicing were also applied to identify subclusters and terminal branches of co-varying features (Figure 3, Figure 4 and Figure 5, Table S2).

#### 2.3.3. Feature-to-Feature Correlation Matrix Including All Features

The feature-to-feature correlation matrix for all 14,883 features across 363 specimens, rather than the post-filtered 5000 features, was computed to identify the most correlated features to further facilitate the subsequent feature annotation process.

#### 2.3.4. Feature Annotations

Selected features were annotated using MS-FINDER version 3.61 [22,23], which predicts candidate molecular formulas and putative chemical structures in silico from MS/MS spectra exported from MS-DIAL. The output may include candidates from Metabolic In silico Network Expansions (MINE) databases, which comprise computationally predicted biochemically plausible metabolites rather than experimentally confirmed compounds [24]. The results of correlation-based hierarchical clustering and the feature correlation matrix were also used to assist the feature annotation process by evaluating potential in-source fragmentation, different adducts, and the biomedical plausibility of the annotation candidates. Annotations were categorized based on the Chemical Analysis Working Group Metabolomics Standards Initiative (MSI) [25] and are summarized in Table 2. Only MSI Level 1 assignments were treated as experimentally confirmed chemical identities; MSI Level 2/3 assignments were considered putative, and MSI Level 4 features remained unknown.

## 3. Results

Among the 14,883 features extracted across all specimens, 138, 121, and 104 features were associated with OPIA-, OXY-, and 6MAM-EIA positivity, respectively, by at least one statistical method. They were designated the corresponding EIA-associated discovery feature sets (Figure 1).

Multiple features were shared among the EIA-associated discovery feature sets (Figure 2). Overlap was smallest between the OXY- and 6MAM-associated discovery feature sets (five features), compared with 14 features shared between the OPIA- and 6MAM-associated discovery feature sets and 36 features shared between the OPIA- and OXY-associated discovery feature sets (Table S1).

We next conducted cluster analyses on the normalized data matrix to classify EIA-associated features into groups, better understand the metabolomic signatures of these groups, and facilitate interpretation of unannotated or partially annotated features. The features were grouped into seven high-level clusters for each EIA result (Clusters A–G for each EIA) (Figure 3, Figure 4 and Figure 5, Table S2).

We also evaluated the top 20 most correlated features based on the feature correlation matrix calculated from all 14,883 features across 363 specimens (Table S3). This analysis supported the feature annotation process by identifying structurally or metabolically related chemicals and analytically related ion signatures, including different ion adducts and in-source ion fragments.

Using these complementary analyses, we evaluated the features selected by at least two statistical methods, with 21, 17, and nine features in the OPIA-, OXY- and 6MAM-associated consensus feature sets. Cluster membership and correlated features were also considered during the annotation process. The annotations, clusters, and MSI confidence levels for each consensus feature set are summarized in Table 3, Table 4 and Table 5.

### 3.1. OPIA-Associated Consensus Feature Set

We evaluated the 21 features selected by more than one analysis for OPIA-EIA (OPIA-associated consensus feature set) (Table 3). These features included oxycodone/opiate metabolites (Cluster E3-1), acetaminophen metabolites (Cluster A1-1), norfentanyl (Cluster D2-2), and others.

**Table 3:** Twenty-one significant features positively associated with opiate-EIA (OPIA-EIA) results selected at least by two analyses (OPIA-associated consensus feature set). Annotations in bold indicate confirmed annotations from spiking studies (MSI Level 1). Other EIAs in bold indicate the EIA-associated consensus feature list, whereas those in regular font indicate the EIA-associated discovery feature list.

k60 Cluster	Feature	Annotation	Other EIAs	Correlation	Volcano	EBAM	SAM	RF
E3-1	318.17166_1.461	**α-oxycodol (C_18_H_23_NO_4_, [M + H]^+^, MSI Level 1)**	**OXY**	X	X			X
E3-1	318.1813_1.637	**β-oxycodol (C_18_H_23_NO_4_, [M + H]^+^, MSI Level 1)**	**OXY**	X	X			X
E1-10	355.17404_7.768	Unknown (MSI Level 4)	OXY	X	X	X	X	
A1-1	313.09753_1.519	**Acetaminophen mercapturate (C_13_H_16_N_2_O5S [M + H]^+^, MSI Level 1)**		X	X	X	X	X
A1-1	328.11508_0.92	**Acetaminophen glucuronide (C_14_H_17_NO_8_, [M + H]^+^, MSI level 1)**		X	X			X
D2-2	233.17049_3.226	**Norfentanyl (C_14_H_20_N_2_O, [M + H]^+^, MSI Level 1)**	**6MAM**	X	X			
E1-10	313.16727_5.187	Unknown (MSI Level 4)	OXY	X	X			
E1-10	371.14999_7.695	Unknown (MSI Level 4)	OXY	X	X			
C1-6	381.07968_0.73	Putative phenolic acid glucuronide derivative (C_15_H_18_O_10_, [M + Na]^+^, MSI level 3)		X	X	X	X	
E3-1	286.15695_1.378	**Composite feature—noroxycodol (C_17_H_21_NO_4_, [M + H − H_2_O]^+^), norcodeine (C_17_H_19_NO_3_, [M + H]^+^), and hydromorphone (C_17_H_19_NO_3_, [M + H]^+^) (MSI level 2, but each component standard confirmed)**	**OXY**	X	X			
E1-10	329.16415_3.802	Unknown (MSI Level 4)		X	X			
A1-1	152.07976_1.055	**Acetaminophen sulfate aglycone (C_8_H_9_NO_2_, [M + H]^+^, MSI level 1)**		X	X	X	X	X
E3-1	241.11029_1.961	Unknown (MSI Level 4)	OXY	X	X			
A1-1	232.03848_0.995	**Acetaminophen sulfate (C_8_H_9_NO_5_S, [M + H]^+^, MSI level 1)**		X	X	X	X	
C1-8	376.13434_1.177	Unknown (MSI Level 4)		X	X			
E1-1	132.04315_1.227	Unknown (MSI Level 4)	**OXY**	X	X	X	X	
A1-1	314.09378_1.466	**Acetaminophen mercapturate isotopologue (C_13_H_16_N_2_O_5_S, [M + H]^+^ isotopic peak, MSI level 1)**		X	X			X
A1-1	271.08298_0.954	**3-(Cystein-S-yl)acetaminophen (C_11_H_14_N_2_O_4_S, [M + H]^+^, MSI Level 1)**			X	X		
A1-1	152.07976_1.422	**Acetaminophen (C_8_H_9_NO_2_, [M + H]^+^, MSI level 1)**			X			X
E2-3	264.08627_1.057	**α-phenylalanylaspartic acid (C_13_H_16_N_2_O_5_, [M + H − NH_3_]^+^, MSI level 1)**	**OXY**		X			X
A1-1	345.14368_0.922	**Acetaminophen glucuronide (C_14_H_17_NO_8_, [M + NH_4_]^+^, MSI level 1)**				X	X	

#### 3.1.1. Oxycodone Metabolites (Cluster E3-1)

Cluster E3-1 contains multiple oxycodone metabolites. These features have been validated by spiking studies and are already included in the routine CUDS reports. These oxycodone metabolites can contribute to OPIA-EIA positivity, although the OPIA-EIA is less sensitive to oxycodone-related compounds than OXY-EIA.

Feature 286.15695_1.378 is likely a composite feature of noroxycodol [M + H − H_2_O]^+^ (RT 1.34 and 1.55), norcodeine [M + H]^+^ (RT 1.60), and hydromorphone [M + H]^+^ (RT 1.20). Other opiates and their metabolites, including morphine (feature 286.15637_0.97), morphine-6-glucuronide (462.18698_0.885), hydrocodone (300.15921_2.15), norhydrocodone (286.14444_2.138), and dihydrocodeine-6-glucuronide (478.21341_1.086) have been filtered out by MetaboAnalyst as less contributory features before application of multiple statistical analyses.

Feature 241.11029_1.961 also belongs to cluster E3-1; however, it does not appear to be a morphinan (morphine/codeine/hydrocodone)-type metabolite. Thus, feature 241.11029_1.961 cannot be associated with oxycodone metabolism, and its chemical identity is unknown.

#### 3.1.2. Acetaminophen Metabolites (Cluster A1-1)

Cluster A1-1 contains multiple acetaminophen metabolites that had been confirmed by spiking studies and were already included in the routine CUDS reports. The features in cluster A1-1 are found only in the OPIA-associated discovery feature set, not in the OXY- or 6MAM-associated discovery feature sets. Because the OPIA-EIA does not cross-react with acetaminophen or its metabolites, their association indicates co-occurrence rather than analytical cross-reactivity.

#### 3.1.3. Fentanyl Metabolite (Cluster D2-2)

Feature 233.17049_3.226 was confirmed as norfentanyl and was already included in the routine CUDS reports. OPIA-EIA does not cross-react fentanyl or norfentanyl, but recreational fentanyl is often co-ingested with heroin (diacetylmorphine), which is metabolized to 6-monoacetylmorphine, morphine, and its metabolites, causing positive OPIA-EIA results.

### 3.2. OXY-Associated Consensus Feature Set

We evaluated the 17 features selected by more than one analysis for OXY-EIA (OXY-associated consensus feature set) (Table 4). These features included oxycodone metabolites (Clusters B3-1, E2-3, and G1-1), dipeptide (α-phenylalanylaspartic acid) (Clusters G3-6 and G3-7), vitamin B6 metabolite (4-pyridoxic acid) (Cluster C2-1), and others.

**Table 4:** Seventeen significant features positively associated with oxycodone-EIA (OXY-EIA) results selected at least by two analyses (OXY-associated consensus feature set). Annotations in bold indicate confirmed annotations from spiking studies (MSI Level 1). Other EIAs in bold indicate the EIA-associated consensus feature list, whereas those in regular font indicate the EIA-associated discovery feature list.

k60 Cluster	Feature	Putative Annotation	Other EIAs	Correlation	Volcano	PLS	EBAM	SAM	RF
E2-3	304.15869_1.362	**Noroxycodol (C_17_H_21_NO_4_, [M + H]^+^, MSI level 1)**	OPIA	X	X	X	X	X	X
E2-3	318.1813_1.637	**β-oxycodol (C_18_H_23_NO_4_, [M + H]^+^, MSI Level 1)**	**OPIA**		X				X
E2-3	318.17166_1.461	**α-oxycodol (C_18_H_23_NO_4_, [M + H]^+^, MSI Level 1)**	**OPIA**		X				X
G1-1	286.15695_1.378	**Composite feature—noroxycodol (C_17_H_21_NO_4_, [M + H − H_2_O]^+^), norcodeine (C_17_H_19_NO_3_, [M + H]^+^), and hydromorphone (C_17_H_19_NO_3_, [M + H]^+^) (MSI level 2, but each component standard confirmed)**	**OPIA**		X				X
C3-7	264.08627_1.057	**α-phenylalanylaspartic acid (C_13_H_16_N_2_O_5_, [M + H − NH_3_]^+^, MSI level 1)**	**OPIA**		X				X
C3-6	281.11124_1.08	**α-phenylalanylaspartic acid (C_13_H_16_N_2_O_5_, [M + H]^+^, MSI level 1)**			X		X	X	
A2-1	153.13197_4.525	Putative monoterpenoid-derived aglycone fragment (C_10_H_16_O, [M + H]^+^, MSI level 3)			X			X	
C3-6	260.06854_1.274	Unknown (MSI Level 4)	OPIA		X	X		X	
C3-1	132.04315_1.227	Unknown (MSI Level 4)	**OPIA**		X			X	
B2-1	164.04114_1.479	Putative N-acetylcysteine-related molecule (C5H9NO3S, [M + H]^+^, MSI level 3)	OPIA		X			X	
E2-3	300.16852_1.543	**Oxycodol (C_18_H_23_NO_4_, [M + H − H_2_O]^+^) or codeine (C_18_H_21_NO_3_, [M + H]^+^) (MSI Level 1)**	OPIA		X	X			
C2-1	184.0605_0.905	**4-Pyridoxic acid (C_8_H_9_NO_4_, [M + H]^+^, MSI level 1)**			X	X			
B1-2	129.10475_0.888	Unknown (MSI Level 4)			X				X
A2-4	541.25806_6.102	Putative glucuronidated metabolite of C21-compound (C_27_H_40_O_11_, [M + H]^+^, MSI level 3)			X	X			
C3-3	172.09702_1.028	Putative N-acyl heterocycle (C_8_H_13_NO_3_, [M + H]^+^, MSI level 3)				X	X	X	X
A2-11	484.30273_8.504	Unknown (MSI Level 4)				X		X	X
B3-1	288.12936_1.054	**Noroxymorphone (C_16_H_17_NO_4_, [M + H]^+^, MSI level 1)**				X			X

#### Oxycodone Metabolites (Clusters B3-1, E2-3, and G1-1)

Cluster E2-3 contains multiple oxycodone reductive metabolites, including oxycodols and noroxycodol. These metabolites had been previously validated and were already included in the routine CUDS reports. In contrast, feature 288.12936_1.054, annotated as noroxymorphone, is assigned to cluster B3-1, whereas feature 286.15695_1.378, a composite feature representing noroxycodol, norcodeine, and/or hydromorphone, is assigned to cluster G1-1. Thus, these features are separated from the main cluster of oxycodone reductive metabolites.

Most of these features are also found in the OPIA-associated consensus feature list, except for feature 288.12936_1.054 (noroxymorphone), presumably because the OPIA-EIA used in this study shows very weak reactivity to noroxymorphone.

### 3.3. 6MAM-Associated Consensus Feature Set

We evaluated the nine features selected by more than one analysis for 6MAM-EIA (6MAM-associated consensus feature set) (Table 5). These features include norfentanyl and 3-hydroxycotinine artifact (cluster B1-5) and 6-acetylmorphine itself (cluster C1-4), all validated by spike studies and already included in the CUDS reports. These analytes are all related to recreational chemical usage.

**Table 5:** Nine significant features positively associated with 6-monoacetylmorphine-EIA (6MAM-EIA) results selected at least by two analyses (6MAM-associated consensus feature set). Annotations in bold indicate confirmed annotations from spiking studies (MSI Level 1). Other EIAs in bold indicate the EIA-associated consensus feature list, whereas those in regular font indicate the EIA-associated discovery feature list.

k60 Cluster	Feature	Putative Annotation	Other EIAs	Correlation	Volcano	RF
B1-2	233.17049_3.226	**Norfentanyl (C_14_H_20_N_2_O, [M + H]^+^, MSI Level 1)**	**OPIA**	X	X	X
C1-4	328.17181_1.974	**6-monoacetylmorphine (C_19_H_21_NO_4_, [M + H]^+^, MSI Level 1)**	OPIA	X	X	
B3-2	141.05748_1.454	Putative methoxyphenol class, unknown isomer (C_7_H_8_O_3_, [M + H]^+^, MSI Level 3)		X	X	
B1-1	238.08284_1.362	Unknown (MSI Level 4)		X	X	X
B3-4	216.12822_1.072	Putative acylcarnitine (C3:1) (C_10_H_17_NO_4_, [M + H]^+^, MSI Level 3)			X	X
C1-3	151.04053_1.44	Putative phenylglyoxylic acid–related metabolite/ion (C_8_H_6_O_3_, [M + H]^+^, MSI level 3)	OPIA		X	X
B1-2	193.24315_0.872	**3-Hydroxycotinine artifact (C_10_H_12_N_2_O_2_, [M + H]^+^, MSI Level 1)**			X	X
E1-5	340.24518_9.444	Unknown (MSI Level 4)			X	X
G1-1	137.06155_3.67	Unknown small aromatic class (C_8_H_8_O_2_, [M + H]^+^, MSI Level 3)			X	X

## 4. Discussion

This retrospective untargeted metabolomics analysis was conducted with routine CUDS data to identify the features associated with opiate exposure using OPIA-EIA, OXY-EIA, and 6MAM-EIA results. The strengths of this study include (i) the large-scale, real-world clinical dataset based on the untargeted urine LC-qToF-MS analyses yielding 14,883 features for 363 routine urine toxicology specimens; (ii) pre-specified, rigorous statistical pipeline applied independently to each EIA endpoint (not post hoc cherry-picking) for systematic feature selection; (iii) correlation-based clustering and MS/MS inspection to prioritize coherent, biologically interpretable feature groups rather than isolated peaks; and (iv) direct translational relevance to everyday clinical toxicology, since all data are derived from routine urine drug screening specimens in a clinical laboratory of an academic medical center in the US.

Feature annotation remains a major challenge in untargeted metabolomics [26,27]. We therefore combined MS-FINDER output, which incorporates accurate mass and MS/MS spectral information [22,23], with a feature correlation matrix and hierarchical clustering to recognize isotopologues, adducts, in-source fragments, and biomedically coherent drug/metabolite groups (Tables S2 and S3). This orthogonal evidence strengthened candidate assignments within biomedically coherent drug/metabolite clusters but did not convert putative annotations into confirmed identities.

### 4.1. Target Metabolites of OPIA-EIA, OXY-EIA, and 6MAM-EIA

The three EIAs used to define opioid exposure represent distinct “exposure selectors” rather than interchangeable opioid markers. The OXY-EIA (100 ng/mL cutoff) is comparatively sensitive and specific for therapeutic oxycodone exposure, whereas the morphine-calibrated OPIA-EIA (300 ng/mL cutoff) has a broader opiate coverage, including hydromorphone, codeine, and 6-monoacetylmorphine; however, the OPIA-EIA requires substantially higher concentrations of oxycodone and oxymorphone (1500 ng/mL and 9300 ng/mL each) to cross-react and become positive. In contrast, the 6MAM-EIA (20 ng/mL cutoff) is highly specific for the heroin-specific metabolite (6-monoacetylmorphine), which reveals very recent heroin exposure. Because of the short detection window of 6-monoacetylmorphine, 6MAM-EIA may capture an acute-use phenotype that differs from the chronic opioid-treated population enriched in OXY-EIA and many OPIA-EIA positive specimens.

### 4.2. Oxycodone Metabolites-Related Features

Oxycodone metabolites are enriched not only in the OXY-associated consensus feature set, but also in the OPIA-associated consensus feature set, likely reflecting a combination of OPIA-EIA broad cross-reactivity and overlap between OPIA-EIA-positive and oxycodone-exposed specimens. Among these metabolites, α-/β-oxycodol was detected in both the OXY- and OPIA-associated consensus feature sets. α-/β-oxycodol is formed through 6-ketoreduction of oxycodone, a metabolic route distinct from the ones mediating the formation of noroxycodone and oxymorphone [28,29,30]. Although α-/β-oxycodol has often been omitted from simplified oxycodone metabolic schemes or treated as a minor reductive metabolite [31,32], its reproducible association with both OXY-EIA and OPIA-EIA positivity highlights its value as another analytically informative biomarker of oxycodone exposure.

The separation of noroxymorphone from the oxycodone reductive metabolite cluster with oxycodol and noroxycodol in the OXY-associated discovery feature set (Table 4) may reflect its distinct metabolic position at the intersection of N-demethylation and O-demethylation pathways by CYP3A and CYP2D6, as well as variability in CYP-mediated metabolism and subsequent conjugation [33,34]. Therefore, noroxymorphone may behave as a less consistent oxycodone-associated marker than the major reductive metabolites in untargeted urine metabolomics. Even though noroxymorphone is chemically identical to nor-naloxone, a naloxone metabolite [35], the absence of naloxone (Feature 328.16931_1.645) within the same cluster B or among the most strongly correlated features (Tables S2 and S3) argues against naloxone exposure as the dominant explanation for feature 288.12936_1.054.

### 4.3. Fentanyl Metabolite-Related Features

Fentanyl is a synthetic opioid with no shared chemical structures with opiates; thus, fentanyl and its major metabolite norfentanyl do not cross-react with the antibodies used in OPIA- and 6MAM-EIA kits. Nevertheless, norfentanyl is found in both the 6MAM- and OPIA-associated consensus feature sets. Given the increasing replacement of heroin by illicitly manufactured fentanyl [3], this pattern is consistent with fentanyl co-exposure among specimens positive for heroin- or morphine-related immunoassays.

### 4.4. Recreational Chemical-Related Features

In addition to the features associated with opioid abuse, the feature related to smoking (e.g., 3-hydroxycotinine artifact) is also found in the 6MAM-associated consensus feature set (Table 5). Furthermore, the 6MAM-associated discovery feature set contains at least six additional smoking-related features (e.g., 193.10092_0.876 for 3-hydroxycotinine, 177.10405_0.988 for cotinine, 179.12364_0.927 for nicotine-N-oxide, 163.12866_0.849 for nicotine), eight cocaine-related features (e.g., 200.12935_0.795 for methyl ecgonine, 304.15652_4.399 for cocaine, 214.15097_0.935 for ethyl ecgonine), and an in-source fragment of methamphetamine (91.05488_2.605), all in cluster B (Table S2). Most of these features were also found in the cocaine-EIA-associated feature set, as reported previously [11]. Overall, recreational chemical-related features are found predominantly among the 6MAM-EIA-associated features.

Some recreational chemical-related features are also found in the OPIA-associated features. In addition to norfentanyl (Table 3), 6-monoacetylmorphine is also included in the OPIA-associated discovery feature set (Table S2). Thus, the OPIA-associated feature set occupies a heterogeneous and intermediate position between 6MAM- and OXY-associated feature sets, shaped by the broad OPIA-EIA reactivity, heroin metabolism into morphine, fentanyl co-exposure, and real-world opioid polysubstance use profile.

Overall, these metabolomic patterns support a dominant exposure gradient model among 6MAM-, OXY-, and OPIA-associated feature sets rather than mutually exclusive exposure classes. In other words, these dominant exposure axes with overlap, rather than three mutually exclusive exposure classes.

### 4.5. Acetaminophen-Related Features

Acetaminophen-related features are only associated with OPIA-EIA, but not with OXY-EIA or 6MAM-EIA. Even though acetaminophen was reported as a cutting agent for heroin before [36], the acetaminophen-related features are not associated with 6MAM-EIA, making a cutting agent improbable as a source of acetaminophen.

Rather, a pharmaceutical source is plausible because acetaminophen is frequently co-formulated or co-administered with opiate analgesics in the United States. In the outpatient drug usage statistics of the United States in 2023, hydrocodone–acetaminophen was the most prescribed opiate analgesic (over 21.5 million prescriptions), followed by oxycodone (over 13.5 million prescriptions), oxycodone–acetaminophen (over 7.2 million prescriptions), morphine (over 3.5 million prescriptions), codeine–acetaminophen (over 2.1 million prescriptions), and hydrocodone (79,852 prescriptions) [37]. It should be noted that acetaminophen co-exposure itself is a data-supported inference; other sources of acetaminophen exposure cannot be fully excluded.

### 4.6. Vitamin B6

Prior metabolomics studies have reported perturbation of vitamin B6-related metabolism in opioid/opiate exposure. In a human urine metabolomics study of Golestan Cohort opium users, pyridoxine and pyridoxal were increased, whereas 4-pyridoxic acid, the major urinary catabolite of vitamin B6, was modestly decreased [38]. Hepatic 4-pyridoxic acid was also decreased in a mouse heroin exposure model [39]. Thus, the positive association between urinary 4-pyridoxic acid and OXY-EIA in the present study appears discordant with the direction of the prior studies.

One plausible explanation is the use of over-the-counter vitamin supplements. OXY-EIA-positive specimens in our clinical cohort may be enriched for patients receiving prescribed oxycodone for chronic pain, and supplement use is common in chronic pain populations [40,41]. And urinary 4-pyridoxic acid reflects recent vitamin B6 intake rather than serving as a specific measure of hepatic vitamin B6 stores [42]. Nevertheless, the present dataset did not include supplement, dietary, renal function, or medication metadata. The 4-pyridoxic acid association should therefore be regarded as hypothesis-generating rather than evidence of an opioid-mediated change in vitamin B6 metabolism.

### 4.7. α-Phenylalanylaspartic Acid

α-phenylalanylaspartic acid is a dipeptide found among the human fecal metabolome [43,44] but, to our knowledge, not previously described as a human urinary metabolite. An isomer β-phenylalanylaspartic acid is a known endogenous dipeptide in human urine and plasma [45], whereas another isomeric dipeptide, α-aspartylphenylalanine, is a metabolic byproduct of aspartame, an artificial sweetener [46]. Even though these isomeric dipeptides have similar MS2 mass spectra consistent with features 264.08627_1.057 and 281.11124_1.08, chemical standard studies showed that α-phenylalanylaspartic acid is the isomer eluting at approximately 1.05 min in our LC-qToF-MS conditions. Thus, these features are annotated as α-phenylalanylaspartic acid (MSI Level 1). To our knowledge, this represents the first report of α-phenylalanylaspartic acid as a urinary metabolomic feature/metabolite in human urine.

The association of α-phenylalanylaspartic acid with OXY-EIA- and OPIA-EIA-positive specimens may reflect altered dipeptide or amino acid metabolism secondary to chronic opioid exposure. Plausible contributors include opioid-induced androgen leading to altered protein turnover in chronic pain populations [47,48,49,50], and possible comorbid renal dysfunction [51,52,53]. But urinary dipeptides can also be influenced by other factors such as diet, nutritional status, or comorbid illness. The present dataset does not contain the clinical metadata needed to evaluate these possibilities. The α-phenylalanylaspartic acid association should therefore be regarded as hypothesis-generating rather than evidence of an opioid-mediated change in dipeptide or amino acid metabolism.

### 4.8. Potential Role of Routine Clinical HRMS Data in Laboratory-Based Drug and Chemical Surveillance

This study was not designed to estimate population prevalence. Nevertheless, LC-qToF-MS data archived from routine clinical toxicology testing could provide a scalable, laboratory-based sentinel framework to detect exposure-associated molecular signatures and to generate early hypotheses about regional drug use and co-exposure trends.

The relevance of this retrospective metabolomics framework for surveillance is further illustrated by the temporal pattern of emerging adulterants in the dataset. For example, the feature corresponding to xylazine (221.12518_3.759, filtered out in the statistical analyses in this study) was included in the datasets collected during 2021–2022, when xylazine was increasingly identified in the U.S. illicit opioid supply [14,54]. In contrast, the features corresponding to medetomidine-related metabolites were not observed. This is also consistent with reports of sporadic detections beginning around 2022–2023 and broader public health concern emerging since 2024 [55,56,57,58].

Accordingly, an archived clinical LC–qToF-MS dataset may provide a chemically resolved historical baseline against which newly emerging drugs and adulterants can be retrospectively queried.

### 4.9. Relationship to Prior Opium/Opiate Metabolomics Studies

Previous metabolomics studies using urine specimens from traditional opium users in the Golestan Cohort Study have provided important reference data for opium- and opiate-associated metabolic perturbations in humans [38,59]. However, the present study differs from the Golestan studies in both study design and intended application. The Golestan studies evaluated community-dwelling adults in Northern Iran, where exposure largely reflected chronic traditional opium use, and opium use or opioid use disorder was defined by self-reported questionnaire or DSM-based interview data. In contrast, our study used clinical specimens from a U.S. hospital-based clinical toxicology population and classified specimens by routine OPIA-, OXY-, and 6MAM-EIA results. Accordingly, Golestan opium users had traditional opium exposure with tobacco-related metabolites and combustion products in Northern Iran, whereas the current study captures prescription opiates (e.g., oxycodone), illicit opioids (e.g., fentanyl, heroin) and various co-exposed substances (e.g., acetaminophen, nicotine, and their metabolites) in the clinical urine specimens in the United States. Thus, the Golestan studies primarily captured metabolic signatures associated with chronic traditional opium exposure, whereas the present study reflects EIA-associated metabolomic signatures of opiate-related and polysubstance exposures encountered in hospital-based urine drug testing in the United States. These studies are therefore complementary; the former provide epidemiologic and mechanistic insight into chronic traditional opium use and opioid use disorder, whereas the present work demonstrates how repurposed clinical LC-qToF-MS data can support biomarker discovery and sentinel surveillance for opiate- and opioid-related drug exposures in real-world clinical specimens.

### 4.10. Validation Priorities and Potential Future Applications

Translation of these findings requires different levels of validation, depending on the feature type. Many xenobiotic features identified in this study, including opiate, fentanyl, and acetaminophen metabolites, have already been analytically confirmed and are reported in routine CUDS. Therefore, their chemical identities do not require further validation. The associations of opiate and fentanyl metabolites with opiate-related EIA positivity largely reflect expected exposure biology and primarily provide internal support for the validity of the analytical workflow. In contrast, the enrichment of acetaminophen metabolites among OPIA-EIA-positive specimens represents a cohort-level co-exposure pattern that may warrant replication in an independent population.

The endogenous features α-phenylalanylaspartic acid and 4-pyridoxic acid require further confirmation of their identities and replication of their associations. However, their clinical utility is not established. Future studies should first determine whether these features provide information beyond routine drug testing results and available clinical data, such as improved characterization of exposure patterns, reproducible metabolic responses, or value for laboratory-based surveillance. Targeted quantitative assay development, analytical reproducibility studies, and implementation assessments would be warranted only if a candidate feature demonstrates such added value. Accordingly, these endogenous findings should currently be regarded as discovery-stage observations rather than clinically actionable biomarkers.

### 4.11. Limitations of This Study

This study has several limitations, some of which were partly addressed previously [11].

First, chemical coverage was limited by using positive ESI mode. Although many drugs are basic [60], urine contains numerous acidic metabolites that ionize weakly under positive ESI conditions [61,62]. Thus, our CUDS LC-qToF-MS dataset predominantly covers basic and neutral chemicals and may underrepresent acidic metabolites.

Second, unsupervised feature filtering prior to MetaboAnalyst-based statistical analysis may have removed biologically informative but sparse features. As the original dataset contained 14,883 aligned features, filtering of low-value or low-variance signals was needed to reduce computational burden and baseline noise [63], but some clinically relevant analytes, including morphine-6-glucuronide and xylazine, were excluded before MetaboAnalyst-based statistical testing. Absence from the final statistically selected feature lists should therefore not be interpreted as absence in the specimens or lack of biological relevance [64].

Third, this was a single-center retrospective study that used EIA results as exposure metadata. The cohort may reflect local prescribing, referral, and illicit drug patterns, and the dataset did not include detailed medication histories, dose or timing, diet, supplementation, renal function, or other clinical covariates. Consequently, the observed associations identify co-varying features but cannot establish causal biological mechanisms or their generalizability to other settings.

Finally, the small number of 6MAM-EIA-positive specimens limits the statistical robustness of the 6MAM-associated findings. Although class imbalance was addressed before random forest analysis using over- and undersampling methods [11], this approach did not increase the number of independent positive specimens. Therefore, the random forest model may be susceptible to overfitting, and its feature importance rankings may be unstable or influenced by cohort-specific polysubstance exposure patterns. Selecting several features using more than one statistical method provides some robustness but does not fully compensate for the limited sample size. Nevertheless, the 6MAM-associated feature sets contained multiple biomedically plausible recreational drug-related features, including 6-monoacetylmorphine, norfentanyl, nicotine-related metabolites, cocaine-related features, and a methamphetamine-related ion. This coherent exposure pattern indicates that the analytical approach captured meaningful biological and toxicological signals despite the limited sample size. Accordingly, even though confidence in the precise feature rankings and generalizability of the model remains limited, the overall 6MAM-associated signature is biologically plausible and supports the validity of the discovery approach.

## 5. Conclusions

In this study, we identified the features associated with opiate exposures using OPIA-, OXY-, and 6MAM-EIA results by retrospective analysis of our CUDS dataset.

OXY-EIA-positive specimens are enriched for prescribed oxycodone exposure, whereas 6-MAM-EIA-positive specimens define a more acute illicit opioid exposure phenotype with stronger polysubstance/recreational use signatures. OPIA-EIA-positive specimens occupy an intermediate and heterogeneous position, shaped by morphine-calibrated immunoassay reactivity, heroin/morphine biology, fentanyl co-exposure, and real-world opioid polysubstance use. These metabolomic patterns indicate a dominant exposure gradient model rather than mutually exclusive exposure classes.

Future work should prioritize further structural annotation of unresolved features in both the discovery and consensus feature sets. The biological, clinical, or surveillance relevance of α-phenylalanylaspartic acid and 4-pyridoxic acid should also be established before targeted assay development is considered. Because 6MAM-positive specimens have become uncommon and our laboratory now uses fentanyl-EIA in place of 6MAM-EIA, direct prospective replication of the 6MAM-associated signature may not be practical. Nevertheless, the archived 6MAM-defined dataset provides a historically informative and biologically coherent example of heroin-associated polysubstance exposure, while future analyses may examine fentanyl-EIA-associated signatures under the current testing paradigm.

## Figures and Tables

**Table 1:** The patient demographics of this study. The number of EIA-positive cases is provided alongside the total case counts by patient location (outpatient or non-outpatient), age, and sex.

Age (Years)	EIA	Outpatient				Non-Outpatient			
		Female		Male		Female		Male	
		EIA+	n	EIA+	n	EIA+	n	EIA+	n
**0–9**	OPIA	0	0	0	0	0	33	0	38
	OXY	0		0		0		0	
	6MAM	0		0		0		0	
**10–19**	OPIA	0	3	0	1	1	30	0	22
	OXY	0		0		0		0	
	6MAM	0		0		0		0	
**20–29**	OPIA	1	10	1	7	1	7	0	10
	OXY	3		2		1		0	
	6MAM	0		0		1		0	
**30–39**	OPIA	3	19	3	17	4	10	2	6
	OXY	3		1		0		0	
	6MAM	1		1		1		2	
**40–49**	OPIA	2	21	5	23	2	6	0	8
	OXY	3		5		1		0	
	6MAM	0		0		0		0	
**50–59**	OPIA	6	15	5	20	0	6	3	13
	OXY	4		3		0		3	
	6MAM	0		0		0		1	
**60–69**	OPIA	1	6	1	12	0	5	0	3
	OXY	1		2		1		1	
	6MAM	0		0		0		0	
**70+**	OPIA	1	2	0	5	0	2	0	3
	OXY	0		0		0		0	
	6MAM	0		0		0		0	
**Total**	**OPIA**	**14**	**76**	**15**	**85**	**8**	**99**	**5**	**103**
	**OXY**	**14**		**13**		**3**		**4**	
	**6MAM**	**1**		**1**		**2**		**3**	

**Table 2:** Annotation terminology and confidence levels used in this study based on the Metabolomics Standards Initiative (MSI) framework.

MSI Level	Terminology	Interpretation	Evidence Used in This Study
Level 1	Identified compound	Chemical identity was confirmed using a chemical standard analyzed under the same analytical conditions	Agreement of retention time and mass-spectral characteristics, including accurate mass and MS2 spectrum, with a chemical standard
Level 2	Putatively annotated compound or composite feature	The possible contributors were individually confirmed using chemical standards, but the observed feature could not be uniquely assigned to a single compound	Chemical standard data supported possible contributors; however, the relative contribution of each compound to the composite feature could not be resolved.
Level 3	Putatively characterized compound class	The available evidence suggested a possible chemical class, structural family, or broad structural type, but did not establish a specific compound identity	Accurate mass, predicted molecular formula, MS2 spectrum, MS-FINDER candidates, retention time, and/or correlation with related features
Level 4	Unknown compound or feature	Requires future structural identification and validation	Reproducible molecular feature without sufficient structural evidence for annotation

## Data Availability

The datasets presented in this article are not readily available because the data are part of an ongoing study. The original datasets generated and/or analyzed during the current study contain protected health information (PHI); thus, they cannot be publicly shared due to the Health Insurance Portability and Accountability Act (HIPAA) Privacy Rule in the U.S.

## Supplementary Materials

The following supporting information can be downloaded at: https://www.mdpi.com/article/10.3390/metabo16080558/s1, Table S1: The feature lists shared among OPIA-, OXY-, and 6MAM-associated discovery feature sets. Table S2: The 138 features in the OPIA-associated discovery feature set, 121 features in the OXY-associated discovery feature set, and 104 features in the 6MAM-associated discovery feature set are grouped into seven high-level clusters, designated as Cluster A through Cluster G, based on hierarchical clustering of their intensity profiles. Within each table, columns represent feature subclusters obtained from middle-level dendrogram slicing, while features enclosed by thin lines indicate terminal branches defined by low-level dendrogram slicing. Table S3: The top 20 most correlated features extracted from the feature correlation matrix for the entire 14,883 features across 363 specimens for each feature in the OPIA-, OXY-, and 6MAM-associated consensus feature sets. The correlation indices are provided within parentheses.

## Abbreviations

The following abbreviations are used in this manuscript:

MS	Mass spectrometry
LC-qToF-MS	Liquid chromatography–quadrupole time-of-flight mass spectrometry
EIA	Enzyme immunoassay
LC-HRMS	Liquid chromatography–high-resolution mass spectrometry
CUDS	Comprehensive urine drug screening
UPMC	University of Pittsburgh Medical Center
ESI	Electrospray ionization
PQN	Probabilistic quotient normalization
SAM	Significance analysis of microarrays and metabolites
EBAM	Empirical Bayesian analysis of microarrays and metabolites
FDR	False discovery rate
PLS-DA	Partial least squares discriminant analysis
RT	Retention time
RF	Random forest
MSI	Metabolomics Standards Initiative
